# Unraveling the intricacies of osteoclast differentiation and maturation: insight into novel therapeutic strategies for bone-destructive diseases

**DOI:** 10.1038/s12276-024-01157-7

**Published:** 2024-02-01

**Authors:** Noriko Takegahara, Hyunsoo Kim, Yongwon Choi

**Affiliations:** Department of Pathology and Laboratory Medicine, University of Pennsylvania Perelman School of Medicine, Philadelphia, PA 19104 USA

**Keywords:** Cell biology, Cell signalling

## Abstract

Osteoclasts are the principal cells that efficiently resorb bone. Numerous studies have attempted to reveal the molecular pathways leading to the differentiation and activation of osteoclasts to improve the treatment and prevention of osteoporosis and other bone-destructive diseases. While the cumulative knowledge of osteoclast regulatory molecules, such as receptor activator of nuclear factor-kB ligand (RANKL) and nuclear factor of activated T cells 1 (NFATc1), contributes to the understanding of the developmental progression of osteoclasts, little is known about how the discrete steps of osteoclastogenesis modify osteoclast status but not the absolute number of osteoclasts. The regulatory mechanisms involved in osteoclast maturation but not those involved in differentiation deserve special attention due to their potential use in establishing a more effective treatment strategy: targeting late-phase differentiation while preserving coupled bone formation. Recent studies have shed light on the molecules that govern late-phase osteoclast differentiation and maturation, as well as the metabolic changes needed to adapt to shifting metabolic demands. This review outlines the current understanding of the regulation of osteoclast differentiation, as well as osteoclast metabolic adaptation as a differentiation control mechanism. Additionally, this review introduces molecules that regulate the late-phase osteoclast differentiation and thus minimally impact coupled bone formation.

## Metabolic adaptation in osteoclasts: a novel approach to bone disease therapy

This study explores the process of bone remodeling, particularly the role of osteoclasts (cells that break down bone) and osteoblasts (cells that build new bone). There is a lack of understanding about the biology of the osteoclast differentiation process (the process by which a cell changes from one type to another), which is vital for maintaining bone health. The scientists reviewed how osteoclast differentiation is regulated, focusing on cell signaling (communication between cells) and metabolic adaptation (how cells change their metabolism to survive). They discovered certain molecules could change osteoclast-mediated bone breakdown without affecting bone formation, potentially serving as treatment targets for diseases that destroy bone. The study concludes that understanding the molecular complexities of late-phase osteoclast differentiation will help improve the treatment and prevention of osteoporosis and other bone-destructive diseases.

This summary was initially drafted using artificial intelligence, then revised and fact-checked by the author.

## Introduction

Skeletal bone is maintained by bone remodeling, in which old and damaged bone is replaced with new bone through continuous cellular processes that are mainly controlled by bone-resorbing osteoclasts and bone-forming osteoblasts^1,2^. During bone remodeling, osteoclast formation is initiated, and a tiny amount of bone is removed, which is then replaced by new bone formed by osteoblasts; after that, the new bone is mineralized. Bone remodeling takes place at sites known as basic multicellular units, which contain all bone cells, including osteoclasts and osteoblasts, and are asynchronously distributed throughout the skeleton^1,2^. Osteoblast-osteoclast interactions are necessary and must be coordinated in time and space to maintain the balance of focal bone remodeling. The communication mechanism by which bone formation follows bone resorption is known as coupling. Researchers have discovered several coupling mechanisms, such as (1) the release of osteoblast precursor recruitment factors and growth-promoting factors, which are stored in large amounts in the bone matrix through osteoclastic bone resorption^3–13^; (2) the secretion of coupling factors by osteoclasts^14–20^; (3) the expression of coupling factors on the osteoclast cell membrane^21–23^; and (4) the production of extracellular vesicles, which contain coupling factors synthesized by osteoclasts^24,25^. Through these mechanisms, osteoclasts signal osteoblast precursors to prepare for bone formation. A functional imbalance in osteoclasts and osteoblasts unbalances coupling, causing abnormal bone homeostasis.

Various diseases and metabolic abnormalities adversely affect bone health. Activation of osteoclastic bone resorption is a common factor in the pathogenesis of bone loss and fractures. Antiresorptive agents such as bisphosphonate and anti-receptor activator of nuclear factor-kB ligand (RANKL) antibodies (denosumab) are used as treatments. Bisphosphonate specifically inhibits farnesyl diphosphate synthase, an enzyme in the cholesterol biosynthesis pathway, in osteoclasts. This inhibition inactivates osteoclasts, decreases their formation, and increases their apoptosis^26,27^. Denosumab is a humanized monoclonal antibody that binds to and inhibits RANKL, blocking osteoclast formation^28–30^. These osteoclast-targeting treatments are efficiently antiresorptive. However, their long-term use compromises bone strength because inhibiting osteoclast formation and activity also inhibits osteoclast-mediated release of coupling factors, thereby inhibiting osteoblast differentiation and not promoting bone formation^31,32^. Since bone health in the adult skeleton depends on the balance of bone remodeling, understanding the biology of osteoclast differentiation is crucial. Targeting only late-phase differentiation while preserving early osteoclast differentiation, which is necessary for coupled bone formation, would aid in establishing a more effective treatment strategy. Multiple studies have demonstrated that osteoclasts that fail to achieve multinucleation, which is a hallmark of osteoclast maturation, retain their osteoclastic phenotype; these osteoclasts continue expressing osteoclast-related markers such as tartrate-resistant acid phosphatase (TRAP) and cathepsin K and exhibit low levels of bone resorption^33–39^. The evidence indicates that targeting osteoclast maturation could selectively control bone destruction, circumventing the negative effects of general osteoblast suppression.

In this review, we will examine the updated regulatory mechanisms of osteoclast differentiation and focus on cell signaling and metabolic adaptation as key differentiation control mechanisms. Additionally, we will describe the molecules needed for osteoclast maturation, which are essential for the process but dispensable for coupled bone formation.

## Osteoclast differentiation and signaling pathways

Osteoclasts are the primary bone-resorbing cells. These cells are highly specific, multinucleated, phagocytic cells of hematopoietic origin and are characterized by distinct features such as a ruffled border that facilitates demineralization and degradation of the bone matrix. Osteoclast differentiation requires two cytokines. The first is macrophage colony-stimulating factor (M-CSF), which is produced by a variety of cells, including lymphocytes and monocytes, and is necessary for the differentiation of hematopoietic stem cells into monocyte/macrophage lineages and the induction of receptor activator of nuclear factor-kB (RANK)^40–44^. The other is RANKL, which is produced mainly by osteoblasts and osteocytes^45,46^ and is essential for promoting and sustaining osteoclast differentiation^47^. During osteoclast differentiation, an osteoclast undergoes various changes, including changes in gene expression patterns, morphology, and metabolic processes. The developmental progression of osteoclasts can be divided into roughly three phases: commitment, maturation, and resorption.

### Commitment

**Fig. 1:**
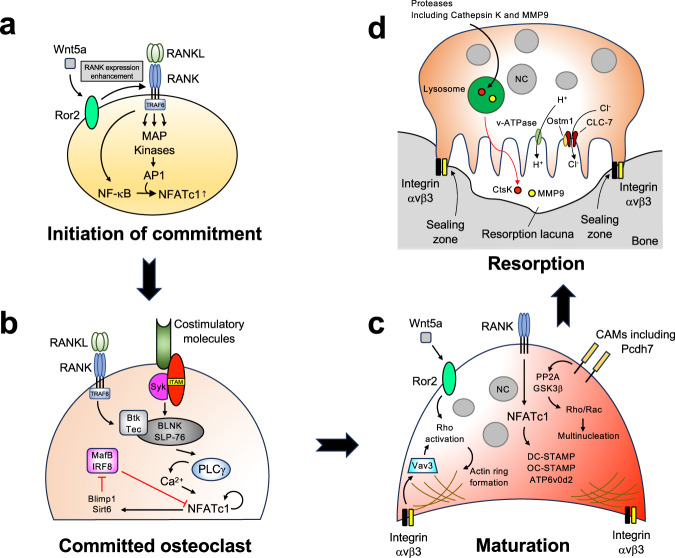
Osteoclast differentiation and signaling pathways. **a** Commitment to osteoclast differentiation is initiated by the engagement of RANKL with RANK. RANK activation recruits adaptor proteins, including TRAF6, thereby initiating the downstream signaling cascade and inducing the expression of osteoclast master regulators. The expression of RANK can be enhanced by additional factors to promote RANKL-induced osteoclast formation. **b** In committed osteoclasts, RANKL-RANK signaling cooperates with costimulatory molecules and promotes PLCγ-mediated Ca^2+^ signaling, which robustly amplifies NFATc1 levels. RANKL-RANK signaling also downregulates the negative regulators of NFATc1. **c** During maturation, committed osteoclasts undergo pronounced morphological changes. Cell adhesion molecules, including integrins and cadherins, regulate the activation of Rho family small GTPases and cytoskeletal organization, contributing to the formation of an actin ring/sealing zone. Additionally, RANKL-RANK signaling induces the expression of osteoclast fusion molecules through the activation of NFATc1, thereby contributing to multinucleation. **d** During resorption, osteoclasts digest inorganic and organic bone matrix by transporting protons and hydrolases through the ruffled border, as well as via endosomal/lysosomal vesicle trafficking. Transcytosis removes the resorbed bone material from the resorption pit. NC nucleus.

The initiation of commitment to the osteoclast lineage from monocyte/macrophage lineage precursors is driven mainly by RANK trimerization and activation^48^ and the recruitment of adaptor proteins, including TNF receptor associated factor 6 (TRAF6), through its intracellular binding sites for the adaptor protein TRAF^49,50^, thereby initiating the downstream signaling cascade. The recruitment of adaptors converges on kinase activation and promotes nuclear translocation and the activation of the critical transcription factors nuclear factor kappa B (NF-κB)^51,52^ and AP1 (consisting of a variety of dimers composed of Fos, Jun, and ATF)^53–55^, which contribute to the initial induction of nuclear factor of activated T cells 1 (NFATc1)^56–60^. NFATc1, NF-κB, and AP1 are the master regulators of osteoclast-specific transcriptional programs. Additional factors, including the noncanonical Wnt ligand Wnt5a^61^, can enhance RANK expression in osteoclast precursors, thereby promoting RANKL-induced osteoclast formation. RANK signaling functions with costimulatory signals via immunoglobulin-like receptor/immunoreceptor tyrosine-based activation motif (ITAM)-motif-containing proteins, such as DNAX-activation protein 12 (DAP12) and Fc receptor gamma-chain (FcRγ)^62^, which associate with the cell surface receptors osteoclast-associated receptor (OSCAR)^63^, paired immunoglobulin-like receptor A (PIR-A)^62^, or triggering receptor expressed on myeloid cells 2 (TREM-2)^64,65^. Activation of costimulatory molecules and tyrosine phosphorylation of ITAM form and activate a complex including spleen tyrosine kinase (Syk), Bruton’s tyrosine kinases (Btk)/Tek, adaptor molecules B-cell linker protein (BLNK), and Src homology 2 domain-containing leukocyte protein of 76 kDa (SLP-76)^66^. This complex integrates both RANK and ITAM downstream signaling, thereby promoting phospholipase Cγ (PLCγ)-mediated Ca^2+^ signaling, which is essential for robust amplification of NFATc1 and its translocation to the nucleus^66^. NFATc1, as well as the transcription factors c-Fos and NF-κB, activates the expression of genes critical to osteoclast activation, including TRAP, cathepsin K, calcitonin receptor, and c-myc^67^. RANKL signaling also downregulates negative regulators of NFATc1, such as v-maf musculoaponeurotic fibrosarcoma oncogene family protein B (MafB)^68^, interferon regulatory factor-8 (IRF-8)^69^, and B-cell lymphoma 6 (Bcl6)^70^. Downregulation of these negative regulators is mediated by the transcriptional repressor B-lymphocyte-induced maturation protein 1 (Blimp1) and sirtuin 6 (Sirt6)^70,71^, both of which are induced by RANKL signaling. The RANKL-RANK signaling pathway orchestrates the expression of these genes, leading committed osteoclasts to progress toward a maturation phase characterized by cell adhesion, fusion, motility, and actin ring formation (Fig. 1a, b).

### Maturation

During maturation, osteoclasts undergo a pronounced morphological change known as multinucleation, which is a hallmark of their maturation. Osteoclasts that cannot achieve multinucleation exhibit diminished bone-resorbing activity, demonstrating the important role of multinucleation in osteoclast function. Multinucleation is a multistep process that is mainly mediated by cell‒cell fusion and repeated incomplete cytokinesis. Committed osteoclasts undergo proliferation^72,73^, migration, cellular adhesion, cytoskeletal rearrangement^74^, and eventually cell‒cell fusion^75^. The transient increase in proliferation induced by RANKL leads to incomplete cytokinesis, contributing to the formation of multinucleated cells^72,73^, which is regulated by Akt activation^73^. The chemoattractant (C-C) ligand-2 (CCL2) and its receptor CCR2 are needed to form multinucleated osteoclasts^76,77^. Cell adhesion molecules (CAMs), such as integrins and cadherins, are needed for committed osteoclasts to adhere to each other and establish close membrane contact and cytoskeletal organization^76,78,79^. CAMs also coordinate RANKL-induced osteoclast differentiation as signal transduction molecules^80,81^. Extensive cytoskeletal rearrangements occur during this phase. Eventually, committed osteoclasts undergo cell‒cell fusion. Several molecules have been shown to be involved in osteoclast fusion, including dendritic cell-specific transmembrane protein (DC-STAMP)^38,82^, osteoclast stimulatory transmembrane protein (OC-STAMP)^36^, and v-ATPase subunit d2 (Atp6v0d2)^83^. NFATc1 and c-Fos, which is downstream of RANKL-RANK signaling, regulates the expression of these fusion-related molecules^56^ (Fig. 1c).

During maturation, osteoclasts adhere to the bone surface, and the resorptive machinery of osteoclasts polarizes toward the bone-cell interface^84^. Signaling mediators, including Rho family small GTPases (RhoA, Rac1, and Cdc42), regulate the extensive cytoskeletal rearrangement that occurs during this phase^85–87^. Integrin αvβ3 mediates the bone-cell interaction and recognizes the arginyl-glycyl-aspartic acid (RGD) sequence present in various bone matrix proteins, such as osteopontin, vitronectin, and bone sialoprotein^88^. The binding of integrin αvβ3 to its ligands phosphorylates and activates c-Src, which phosphorylates Syk in an ITAM-containing protein (DAP12 and FcRγ)-dependent manner^89^. Activated Syk regulates guanine nucleotide exchange factors (GEFs), including Vav3^90^, which eventually activates Rho family small GTPases and regulates cytoskeletal organization. A mature osteoclast eventually forms F-actin-rich adhesive structures, which form a sealing zone (actin ring) on the ventral membranes in contact with the bone surface. This sealing is designed to release protons and proteases for demineralization and to break down the bone matrix^91^. Syk and c-Src are critical for sealing zone formation and bone resorption^89,92,93^ (Fig. 1c).

### Resorption

The functional resorption phase involves the formation of a ruffled border surrounded by the sealing zone^84^. The ruffled border is a highly folded and invaginated membrane structure that increases the surface area of osteoclasts in contact with bone^47^. Through this border, protons and hydrolases that solubilize and digest inorganic and organic bone matrix are transported, enabling mature osteoclasts to efficiently resorb bone. Initially, osteoclasts produce hydrochloric acid, which dissolves the bone mineral. The α3 subunit of v-ATPases, the Cl^-^/H^+^ antiporter chloride voltage-gated channel 7 (CLC-7), and its β-subunit osteopetrosis associated transmembrane protein 1 (OSTM1) localize to the lysosome and the ruffled border, where they acidify secretory lysosomes and the space between the ruffled border and the bone surface^94–97^. This local acidosis dissolves inorganic minerals such as calcium, exposing organic matrix components such as collagen in the connective bone tissue. The decalcified organic matrix is subsequently degraded by lysosomal proteases such as collagenases, cathepsin K, and matrix metalloproteinases (MMPs)^98,99^. The secretory lysosome pathway, which delivers proteases, is regulated by Rab7, a small GTPase that regulates endosomal/lysosomal vesicle trafficking^100^. Synaptotagmin VII, which is a member of the synaptotagmin family that mediates the Ca^2+^-triggered fusion of cytoplasmic/synaptic vesicles to the plasma membrane, mediates the fusion of secretory lysosomes with the ruffled border^101^. The autophagy-related protein Atg5 also participates in the secretion of lysosomal contents by directing lysosomes to fuse with the plasma membrane^102^. Mutations in genes involved in matrix demineralization and dissolution account for most human cases of osteopetrosis^103^. The resorbed material is removed from the resorption pit by transcytosis through osteoclasts^104,105^ (Fig. 1d).

## Osteoclast differentiation and metabolic adaptation

The differentiation of osteoclasts and their bone-resorbing function are energy-intensive processes. Along with signaling pathways and osteoclast-specific gene expression programs, osteoclast differentiation activates metabolic programs. While our understanding of these pathways and gene expression programs during osteoclast differentiation has significantly advanced, the investigation of energy metabolism and its regulation in osteoclasts is in its infancy. However, recent evidence shows that osteoclasts display remarkable metabolic adaptation during differentiation, highlighting the importance of cell metabolism as a differentiation control mechanism (Table 1).

**Table 1:** Key molecules involved in osteoclast differentiation and metabolic adaptation.

Molecule	Function in osteoclasts	References
PGC1β	Regulating mitochondrial biogenesis	^107^
Tfam	Regulating mitochondrial DNA metabolism and mitochondrial biogenesis	^113^
Ndufs4	An accessory subunit of mitochondrial respiratory chain complex I	^117^
Myc	Regulating mitochondrial respiratory capacity	^116^
PKM2	Regulating glycolysis. Downregulation is required for late phase osteoclast differentiation	^123^
HIF1α	Regulating gene expression (especially glycolysis-involved genes) in response to hypoxic stimuli. Important under pathological conditions	^106,133^
COMMD1	Negatively regulating osteoclast differentiation, which is inhibited by hypoxia. Important under pathological conditions	^124^

### Oxidative phosphorylation and osteoclast numbers

**Fig. 2:**
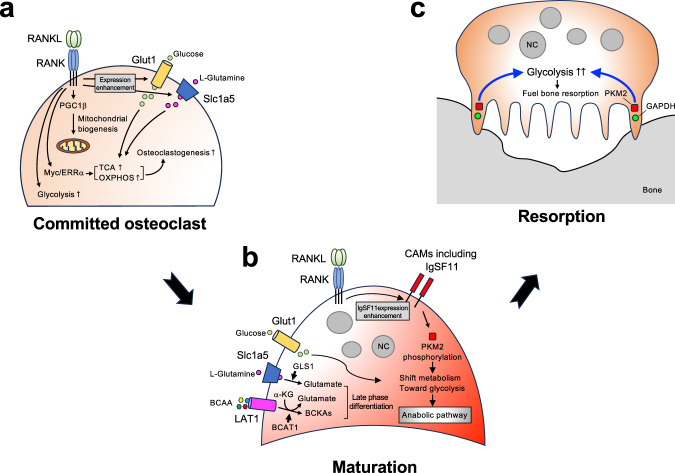
Osteoclast differentiation and metabolic adaptation. **a** In committed osteoclasts, RANKL-RANK signaling enhances mitochondrial biogenesis, which is regulated in part by PGC1β. In addition to increasing mitochondrial biogenesis, RANKL stimulation upregulates TCA cycle- and oxidative phosphorylation-related genes, enhances mitochondrial respiration, and results in the production of high levels of ATP. The Myc-ERRα signaling axis mediates this process. Oxidative phosphorylation is needed for osteoclast differentiation, thereby contributing to osteoclast numbers. Moreover, RANKL-RANK signaling enhances glycolysis. **b** Mature osteoclasts have a high glycolytic rate. RANKL-RANK signaling enhances the expression of IgSF11, which regulates the activity of the glycolysis rate-limiting enzyme PKM2. Consequently, glucose metabolism shifts toward an anabolic pathway rather than energy production. Moreover, alternative energy substrates, such as glutamine and BCAAs, fuel osteoclast differentiation. **c** Glycolysis fuels bone resorption. The glycolysis-associated enzymes PKM2 and GAPDH were detected in sealing zones. NC nucleus.

Osteoclast differentiation is associated with a substantial increase in biomass^106^. High metabolic activity and adenosine triphosphate (ATP) production are required for these processes. The energy produced by a cell mainly depends on glucose. Glucose is used to produce ATP through the glycolysis pathway, which occurs in the cytoplasm, and the tricarboxylic acid cycle (TCA cycle) and oxidative phosphorylation, both of which occur in mitochondria. Several studies have suggested that both oxidative phosphorylation and glycolysis are increased during osteoclast differentiation. When osteoclast precursors receive RANKL signals, mitochondrial biogenesis and, consequently, mitochondrial size and numbers increase^107,108^. The increase in oxidative phosphorylation is mediated at least in part by an increase in mitochondrial biogenesis orchestrated by peroxisome proliferator-activated receptor-g coactivator 1β (PGC1β)^107^. Mitochondrial DNA is critical for mitochondrial biogenesis, and disruptions in mitochondrial transcription factor A (Tfam) cause severe respiratory chain deficiency and reduced ATP production^109–112^. Tfam deficiency in osteoclasts significantly reduces osteoclast numbers^113^. However, the prerequisite of mitochondrial biogenesis for osteoclast differentiation and activity remains in question, as osteoclast lineage-specific PGC1β-deficient mice have a normal bone phenotype, and Tfam-deficient osteoclasts have increased resorption activity and accelerated apoptosis despite the significant decrease in mitochondrial biogenesis levels^113,114^. Along with increased mitochondrial biogenesis, RANKL stimulation upregulates the expression of the genes involved in the TCA cycle and oxidative phosphorylation^108,115,116^. This accelerates mitochondrial respiration and produces high levels of ATP. Oxidative phosphorylation has been identified as the primary bioenergetic source for osteoclast formation. NADH:ubiquinone oxidoreductase iron-sulfur protein 4 (Ndufs4) is a subunit of mitochondrial membrane respiratory chain complex I. Ndufs4 deficiency suppresses mitochondrial complex I activity, inhibiting osteoclast differentiation and thereby increasing bone mass in vivo^117^. The RANKL-induced increase in the expression of oxidative phosphorylation-involved genes and enhanced mitochondrial respiration depend on the Myc-ERRα signaling axis^116^. In addition to oxidative phosphorylation, S-adenosylmethionine (SAM)-mediated DNA methylation by Dnmt3a regulates osteoclast differentiation via epigenetic repression of anti-osteoclastogenic genes, including IRF-8^115^. Myc deficiency in osteoclasts severely impairs mitochondrial respiratory capacity and completely stops osteoclastogenesis, thereby increasing bone mass in vivo^116^. These observations strongly indicate that oxidative phosphorylation is required for osteoclast differentiation. Moreover, disturbing oxidative phosphorylation in osteoclasts changes the bone phenotype, specifically by decreasing osteoclast numbers (Fig. 2a).

### Glycolysis, osteoclast maturation and resorption

Glycolysis is the metabolic pathway that oxidizes glucose for energy production and anabolic processes such as protein, lipid, and nucleic acid synthesis and supports proliferation and growth^118–120^. RANKL stimulation induces the expression of glycolysis-involved genes, including hexokinase (HK), phosphofructokinase (PFK), and pyruvate kinase (PKM), which are rate-limiting enzymes that control the pace of glycolysis and the outflow from the glycolytic pathway to the TCA cycle^106^. RANKL also induces lactate dehydrogenase (LDH), which catalyzes the transformation of pyruvate into lactate^121^, as well as the glucose transporter Glut1^106^. During RANKL-induced osteoclast differentiation, glucose consumption and lactate production are elevated, indicating an increase in glycolysis^122,123^. The importance of glycolysis for osteoclast differentiation has been demonstrated using specific inhibitors and/or activators. The molecule 2-deoxy-D-glucose (2-DG) is a modified glucose molecule with the 2-hydroxyl group replaced by hydrogen. This factor accumulates in the cell and inhibits glucose phosphorylation by HK, preventing further glycolysis. Treatment with 2-DG severely abrogates osteoclast differentiation^116,124^. PKM isoform 2 (PKM2) is a rate-limiting glycolysis enzyme whose activity can be negatively regulated^118,125^. PKM2 catalyzes the conversion of phosphoenolpyruvate to pyruvate. Active PKM2 contributes to glucose metabolism by directing it toward mitochondrial oxidative phosphorylation, thereby increasing ATP production through the mitochondrial respiratory chain. Conversely, when PKM2 is negatively regulated, glucose metabolism shifts toward aerobic glycolysis. Thus, glucose metabolites are increased and directed toward anabolic pathways^120,126^. Activation of PKM2 using the specific activator TEPP46 reduces glycolysis (i.e., shifts glucose metabolism toward oxidative phosphorylation) and inhibits the formation of large multinucleated osteoclasts, whereas PKM2 inhibition by the specific inhibitor shikonin enhances osteoclast differentiation^123^. Phosphorylation of PKM2 at tyrosine 105 (Y105) can negatively regulate its enzymatic activity^125^. Increased phosphorylation of PKM2 Y105 is observed during RANKL-induced osteoclast differentiation, especially in the late phase (maturation)^123^. These results suggest that glycolysis plays a key role in osteoclast differentiation, especially during maturation. Glycolysis is also an influential factor in the bone resorption phase. Osteoclastic resorption is significantly decreased when osteoclasts are fueled by galactose instead of glucose, which forces cells to depend on oxidative phosphorylation to generate sufficient ATP by reducing the glycolysis rate^108^. The impaired resorption activity in the presence of galactose can be restored with a nontoxic dose of rotenone, an inhibitor of mitochondrial complex I^108^. These observations indicate that glycolysis is the preferred energetic pathway driving the resorption activity of mature osteoclasts. Notably, localization of the glycolysis-associated enzymes PKM2 and glyceraldehyde-3-phosphate dehydrogenase (GAPDH) has been detected in proximity to the sealing zones of mature osteoclasts^108^. This observation supports the idea that glycolysis fuels the bone resorption process (Fig. 2a–c).

Oxygen tension is very low in bone, especially in resorption lacunae, where osteoclasts reside. Therefore, oxygen availability may dictate the metabolic profile and modulate osteoclast formation and activation. Hypoxia is a critical stimulator of osteoclast differentiation^124^, although conflicting data exist^127^. The hypoxia-inducible transcription factor HIF is the main regulator of the hypoxic response and plays a crucial role in osteoclast regulation. HIF is a heterodimer composed of an inducible alpha subunit (HIF-1α and HIF-2α) and a constitutively expressed beta subunit (HIF-1β). In hypoxic microenvironments, HIF proteins are stabilized, establishing an active transcriptional complex. Hypoxia stabilizes HIF-1α in osteoclasts^128–132^, and RANKL-induced osteoclast differentiation increases HIF-1α expression^128,133^. HIF-1α activation induces the transcription of Glut1 and rate-limiting glycolytic enzymes such as lactate dehydrogenase A (LDHA) and phosphofructokinase liver type (PFKL), thereby promoting glucose uptake and glycolysis in osteoclasts^106^. Inhibiting HIF-1α significantly inhibits osteoclastic resorption despite enhancing the formation of large multinucleated osteoclasts^106^. Hypoxia also augments osteoclast differentiation by increasing the glycolysis metabolic pathway by suppressing the negative regulator copper metabolism MURR1 domain-containing 1 (COMMD1)^124^. Importantly, osteoclast-specific HIF-1α deficiency or COMMD1 deficiency in mice or treatment with the HIF-1α inhibitor 2-methoxyestradiol results in little change in bone volume or osteoclast numbers on the bone surface under normal conditions^124,133^. However, under conditions of osteoclast activation, such as estrogen deficiency and inflammation, bone loss is prevented by suppressing osteoclast activation^124,133^. These observations indicate the important role of HIF-1α and COMMD1 (and possibly the molecule-dependent increase in glycolysis) under pathological rather than physiological conditions.

### Alternative energy substrates that fuel osteoclast differentiation

Although glucose is a major nutrient for osteoclasts, limiting glycolysis by omitting glucose from the culture media or knocking down HIF-1α does not impair osteoclast formation^106^, indicating alternative energy substrates that support the differentiation process. During osteoclast differentiation, osteoclasts take up a considerable amount of amino acids, including glutamine, arginine, serine, and branched-chain amino acids (BCAAs)^134^. Glutamine is the most bountiful and flexible amino acid in the body. L-glutamine significantly impacts osteoclast differentiation, and its depletion abrogates the induction of osteoclast formation^106^. Supplementation with L-glutamine during the later phase of differentiation has more impact than during the early phase^106^, indicating its significant role in the later phase of differentiation. The high-affinity Na^+^-dependent L-glutamine transporter solute carrier family 1 member 5 (Slc1a5) mediates L-glutamine uptake, which is catalyzed to form glutamate by glutaminase (Gls1). RANKL stimulation induces both Slc1a5 and Gls1 in osteoclasts. Although the metabolism of glutamate in osteoclasts remains unclear, it is thought to be converted to α-ketoglutarate (α-KG) and subsequently fuels other metabolic pathways, such as the TCA cycle, as a substrate of anaplerosis during osteoclast differentiation^135^. Arginine, a conditionally essential amino acid, has been shown to control RANKL-induced osteoclast formation. Extracellular arginine supports TCA cycle activity and oxidative phosphorylation^136^. Depletion of arginine by recombinant arginase 1 during differentiation decreases RANKL-induced expression of NFATc1 and Fos at an early timepoint and completely blocks osteoclastogenesis^136^. BCAAs and branched-chain aminotransferase 1 (BCAT1) play important roles in osteoclast differentiation. BCAT1 is the cytoplasmic BCAT isoform that converts BCAAs into branched-chain ketoacids, generating glutamate from α-KG^137^. BCAT1 deficiency in mice increases bone mass by decreasing osteoclast differentiation and resorption activity^138^. Pharmacological inhibition of BCAT1 prevents osteoclast differentiation and inflammation-induced bone loss^139^. The depletion of BCAAs during the later phase of differentiation significantly decreases osteoclast maturation, whereas depletion in the early phase has no effects on osteoclast differentiation or maturation^139^, indicating the role of BCAAs in the relatively late phase of osteoclast differentiation. Moreover, conflicting data on the role of BCAAs in osteoclast differentiation arise from osteoclast-specific deletion of LAT1, an L-type amino acid transporter 1 that mediates the uptake of BCAAs (also known as Slc7a5), resulting in enhanced osteoclast differentiation and function, which are attributed to impaired mTOR signaling^140^. Further studies are needed to clarify the impact of BCAA metabolism and BCAA uptake on osteoclast differentiation (Fig. 2a, b).

## Molecules regulating late-phase osteoclast differentiation with minimal impacts on coupled bone formation

Studies have identified molecules whose functional disturbance causes defective osteoclastic bone resorption without affecting osteoblastic bone formation. These molecules can be promising targets to aid in establishing a more effective treatment strategy for late-phase osteoclast differentiation while preserving coupled bone formation (Table 2).

**Table 2:** Key molecules involved in osteoclast late-phase differentiation with a minimal impact on coupled bone formation.

Molecule	Function in osteoclasts	References
BTK/Tec	Integrating RANK and ITAM signaling and promoting PLCγ-mediated Ca^2+^ signaling. Essential for osteoclast differentiation	^66^
Ror2	Promoting osteoclast differentiation by enhancing the expression of RANK in osteoclast precursors. Required for osteoclast differentiation	^61,142^
Pkn3	Enhancing c-Src activity. Required for osteoclastic bone resorbing activity	^142,143^
Pcdh7	Regulating small GTPases. Required for osteoclast multinucleation	^81,144,145^
IgSF11	Regulating PKM2 activity. Required for osteoclast differentiation	^80,123^
PKM2	Regulating glycolysis. Downregulation is required for late phase osteoclast differentiation	^123^

### Btk and Tec are critical signaling molecules for osteoclast differentiation

The tyrosine kinases BTK and Tec are critical for integrating RANK and ITAM downstream signaling and promoting PLCγ-mediated Ca^2+^ signaling^66^. Osteoclasts derived from Xid mice, which possess a natural mutation in the BTK gene, exhibit impaired multinucleated osteoclast formation in vitro due to impaired fusion of preosteoclasts^141^, although no significant defects were detected in BTK-deficient mice in terms of osteoclast numbers, resorption activity, and osteoblastic bone formation. However, Tec and BTK double-deficient mice exhibit striking defects in osteoclast numbers and bone resorption^66^. Notably, osteoblastic bone formation remains intact in double-deficient mice^66^. Local administration of the Tec kinase inhibitor LFM-A13 attenuates inflammation-induced bone loss or RANKL-induced bone loss by decreasing osteoclast numbers on the bone surface but does not affect inflammatory responses^66^. These observations suggest that targeting Tec family kinases can suppress osteoclastogenesis while preserving coupled bone formation in vivo.

### PKN3 is a signaling mediator of the Wnt5a-Ror2 signaling pathway

Cytoskeletal reorganization in osteoclasts to form sealing zones is imperative for the attachment of these cells to bone and the resorption of bone matrices. Wnt5a and receptor tyrosine kinase-like orphan receptor 2 (Ror2) signaling promote osteoclast differentiation by enhancing the expression of RANK in osteoclast precursors by activating c-Jun N-terminal kinase (Jnk)^61^. Additionally, Wnt5a-Ror2 signaling enhances osteoclastic bone-resorbing activity in mature osteoclasts through protein kinase N3 (Pkn3)^142^. Mechanistically, the engagement of Wnt5a with Ror2 stimulates the small GTPase Rho and its effector kinase Pkn3 and eventually enhances c-Src activity. Osteoclasts that lack Ror2 and/or Pkn3 expression have impaired actin ring formation^142^. Heterozygous mutations in Ror2 increase bone volume due to decreased numbers of osteoclasts on the bone surface, and osteoblastic bone formation is normal^61^. Pkn3 deficiency in mice increases bone mass due to impaired osteoclastic bone resorption activity, which is characterized by decreases in the eroded surface and erosion depth on the bone surface, but not by osteoclast differentiation, as the number of osteoclasts on the bone surface is comparable to that in control mice^142^. Osteoblastic bone formation is intact in the absence of Pkn3^142^. These results suggest that Ror2 is required for osteoclast differentiation, whereas Pkn3 is dispensable for osteoclast differentiation but essential for the maturation and resorption activity of osteoclasts. Of note, both Ror2 and Pkn3 are dispensable for coupled osteoblastic bone formation^61,142^. Recently, the Pkn3 inhibitor SB202190 has been shown to inhibit osteoclast activity but not osteoclast differentiation and coupled bone formation in vivo, thereby preventing ovariectomy-induced bone loss^143^. These findings indicate that targeting Pkn3 with SB202190 can be a promising treatment. These findings also suggest that the signaling pathway that regulates the formation of a sealing zone can be a target of a novel anti-bone resorption therapy.

### Pcdh7 is a signal transduction molecule for the activation of small GTPases

Osteoclast differentiation requires costimulatory molecules in addition to the signals of RANKL. Protocadherin-7 (Pcdh7) is a member of the protocadherin family, which is a subgroup of the cadherin superfamily. This factor acts as a signaling receptor for osteoclast differentiation^81,144,145^. Mechanistically, Pcdh7 ligation, which is thought to occur through homophilic interactions, activates protein phosphatase 2 A (PP2A) during RANKL-induced osteoclast differentiation. PP2A dephosphorylates and activates GSK3β, which, in turn, activates small GTPases, including RhoA, inducing osteoclast differentiation and multinucleation. Pcdh7 deficiency in mice reduces osteoclast numbers, thereby increasing bone mass^144^. Pcdh7 deficiency does not affect coupled bone formation^144^. Of note, Pcdh7 deficiency impairs the formation of large multinucleated osteoclasts, although it does not affect the expression of differentiation markers such as Acp5 and Cathepsin K or osteoclastic resorption functions^144^. Pcdh7 may be required during the osteoclast maturation phase to form large multinucleated osteoclasts. These findings suggest that multinucleation is a potential target for regulating osteoclastic bone resorption without affecting coupled bone formation.

### IgSF11 and PKM2 regulate glycolysis

Glucose metabolism, including glycolysis and oxidative phosphorylation, is accelerated during osteoclast differentiation, and it exhibits potential as a target for the regulation of osteoclastic bone resorption. The glycolysis rate-limiting enzyme PKM2 regulates osteoclast differentiation as a signal mediator that is downstream of immunoglobulin superfamily 11 (IgSF11)^80,123^. Stimulation of IgSF11, which is mediated by homophilic interactions, phosphorylates PKM2 at Y105 through multiple src family kinases, including c-Src, thereby inhibiting PKM2 activity. RANKL stimulation induces expression of IgSF11, which peaks during the late phase of differentiation, and tyrosine phosphorylation of PKM2 also peaks during the late phase of differentiation. PKM2 phosphorylation and lactate production during osteoclast differentiation are decreased in the absence of IgSF11, suggesting IgSF11-mediated regulation of PKM2 activity and, thereby, glycolysis^123^. IgSF11 deficiency impairs osteoclast differentiation and resorption activity without affecting early-phase signaling pathways. Consistently, IgSF11 deficiency increases bone mass due to decreased numbers of osteoclasts on the bone surface^80^. Additionally, systemic administration of the PKM2 activator TEPP46 increases bone mass due to decreased numbers and sizes of osteoclasts on the bone surface^123^. IgSF11 deficiency and/or the administration of TEPP46 do not affect coupled bone formation^80,123^. Notably, the inhibitory effect of TEPP46 on osteoclast differentiation is more prominent under inflammatory conditions and do not affect inflammation or coupled bone formation^123^. These findings indicate that IgSF11-PKM2 signaling axis-mediated regulation of glucose metabolism is a promising target for selectively inhibiting bone loss. More importantly, metabolic adaptation is a mechanism controlling osteoclast differentiation and has potential as a target for the treatment and prevention of bone-destructive diseases.

## Conclusions

In this review, we provided an overview of cell signaling and metabolic adaptations as regulatory mechanisms of osteoclast differentiation and specifically focused on understanding these mechanisms in the context of the osteoclast differentiation phases. Through the use of gene-edited mice, we have gained valuable insights into the phases of osteoclast differentiation and identified regulatory molecules that can modulate osteoclast-mediated bone resorption without affecting coupled bone formation. These include (1) the multinucleation process, as characterized by gene deletion of Pcdh7; (2) sealing zone (actin ring) formation, as characterized by Pkn3 deficiency; and (3) RANKL-induced glycolysis, as characterized by the modulation of PKM2 activity. Note that caution should be exercised due to the potential involvement of these molecules in other tissues. Indeed, in mice, Pkn3 deficiency could impact angiogenesis^146^, and bioinformatic analysis identified Pcdh7 as a key gene related to the development of sarcopenia and osteoporosis^147^. Understanding the molecular intricacies underlying late-phase osteoclast differentiation, in addition to its physiological roles, will assist in improving the treatment and prevention of osteoporosis and other bone-destructive diseases.

It is important to acknowledge that while many studies have explored the impact of gene functions on osteoclast differentiation and activity, they mainly focused on the role of genes in intrinsic cell functions. As the coupling between osteoclasts and osteoblasts can only be observed in vivo, some molecules that can potentially impact late-phase osteoclast differentiation (i.e., maturation) without affecting osteoblastic bone formation might have been overlooked. To gain a comprehensive understanding, future studies should reinvestigate the function of these molecules from the perspective of their involvement in coupling mechanisms. This will pave the way for further advancements in the field and potentially unveil novel therapeutic targets for bone-destructive diseases.
